# Correction: Tracheostomy Management in Oral and Oropharyngeal Carcinoma Patients: A Retrospective Study from a Multidisciplinary Protocol Approach

**DOI:** 10.1007/s12070-025-06125-9

**Published:** 2025-10-18

**Authors:** Dhanush, Aseem Mishra, Komal Lamba, Sunayana R Sarkar

**Affiliations:** 1 Department of Head and Neck Surgical OncologyMahamana Pandit Madan Mohan Malviya Cancer Centre and Homi Bhabha Cancer Hospital, Tata Memorial Centre, Varanasi, Uttar Pradesh, India; 2 Homi Bhabha National Institute, Mumbai, Maharashtra, India

**Correction: Indian Journal of Otolaryngology and Head & Neck Surgery**

10.1007/s12070-025-06047-6

In this article, the author’s name Dhanush was incorrectly written as Dhanush Dhanush.

The original article has been corrected.

